# Comparative Analysis of Two Surgical Treatment Options for Giant Cell Tumor of the Proximal Femur: Extended Curettage and Segmental Resection

**DOI:** 10.3389/fonc.2021.771863

**Published:** 2021-12-20

**Authors:** Yuhao Yuan, Qing Liu, Yupeng Liu, Ziyi Wu, Wei Zhong, Hongbo He, Wei Luo

**Affiliations:** ^1^ Department of Orthopaedics, Xiangya Hospital, Central South University, Changsha, China; ^2^ National Clinical Research Center for Geriatric Disorders, Xiangya Hospital, Changsha, China

**Keywords:** proximal femur, giant cell tumor of bone, extended curettage, segment resection, surgical options

## Abstract

**Aim:**

As a locally destructive intermediate bone tumor with low incidence, high recurrence rate, and difficulty in reconstruction, giant cell tumor of bone (GCTB) in the proximal femur has no unified surgical treatment standard. This study aimed to compare the differences in local recurrence, reconstruction durability, and postoperative function after treatment with either extended curettage (EC) or segmental resection (SR) for GCTB in the proximal femur so as to provide constructive suggestions for the rational selection of EC or SR operation scheme.

**Patients and Methods:**

29 patients (15 men and 14 women) were included in this retrospective study, with a mean age of 32.1 years. According to the division method of proximal femur of International Society Of Limb Salvage (ISOLS), there was 1 case in the H1 area, 17 cases in the H2 area, 10 cases in the H1+H2 area, and 1 case in the H1+H2+H3 area. Among them were 11 cases of Campanacci grade II GCTB, 18 cases of Campanacci grade III GCTB, and 7 cases with pathological fractures. All patients underwent either EC or SR surgery. The Musculoskeletal Tumor Society (MSTS) score was used for patient evaluation. The operation effectiveness was analyzed according to the Mankin evaluation standard. Regular follow-up was performed to evaluate the recurrence rate, limb function, and long-term complications of the two surgical methods.

**Results:**

All patients were followed up for a mean of 60.4 months. Local recurrence occurred in one of 19 patients treated with EC (5.3%) and one of 10 patients treated with SR (10%). The MSTS score of lower limb function in patients in the EC group was better compared to patients in the SR group (P = 0.002). Complications occurred in 2 cases (10.5%) and 5 cases (50%) in the EC group (osteoarthritis, osteonecrosis) and SR group (joint stiffness, infection, prosthesis loosening), respectively, with significant differences between the two groups (P = 0.03). The operation effectiveness was analyzed according to the Mankin evaluation standard. The EC group showed an optimal rate of 94.7% (18/19) as opposed to 80% (8/10) in the SR group.

**Conclusions:**

For GCTB in the proximal femur, when the tumor does not extensively involves the surrounding soft tissues, the articular surface was not damaged, and there is no pathological fracture with apparent displacement, EC surgery should be fully considered.

## Introduction

Giant cell tumor of bone (GCTB) is a common primary bone tumor and possesses characteristics of unpredictable biological behavior, severe bone erosion, and a high recurrence rate (1). Studies have shown that GCTB accounts for about 20% of all benign bone tumors, with malignant transformation occurring in about 10% of GCTB and lung metastasis occurring in 1% to 4% of patients. The age of onset is mainly between 20 and 40 years old, women are more common (2). In addition, it is defined as a locally destructive intermediate bone tumor due to its strong bone and soft tissue invasiveness. The epiphyseal regions of the distal femur and proximal tibia are the most common sites, accounting for about 60% - 70% of GCTB in all body parts (3). However, the prognosis varies according to the anatomical site of GCTB. Hence the study of GCTB in different anatomical parts is a must (2, 3).

The incidence rate of GCTB in the proximal femur is relatively low, accounting for only about 5.5% of GCTB. Still, it has the features of a high recurrence rate and poor prognosis (4). The lesions are mainly located in the femoral neck and intertrochanteric. As this region is an essential mechanical conduction pathway of the human body, the probability of pathological fracture is higher than that of GCTB around the knee joint. Although fewer cases can extend to the joint cavity, they can penetrate the subchondral bone and seriously affect the function of the hip joint (5). Besides, considering the blood supply, osteonecrosis is more likely to occur in the progression and treatment of proximal femoral GCTB. Furthermore, previous studies have identified that the postoperative local recurrence is more frequent with a high complication rate of proximal femoral GCTB (6–8). These factors lead to the tortuous dilemma in the treatment of proximal femoral GCTB. The aim of our treatment of proximal femoral GCTB at this stage is primarily to completely remove the lesions, reduce the recurrence rate, restore the flatness of the joint surface and prevent complications. These will help restore the normal biological function of the hip joint to the greatest extent and achieve a satisfactory survival prognosis. Therefore, the treatment of proximal femoral GCTB is more challenging. At present, there are few literature reports on proximal femoral GCTB, and there is no unified treatment principle (9). The choice of surgical methods is also controversial, which mainly include extended curettage (EC) and bone cement filling, segmental resection (SR), and tumor hip prosthesis reconstruction (10, 11). Although both treatments can achieve satisfactory results, the prognosis is inconsistent in the reviews, and each has its advantages and drawbacks. The former can preserve the articular surface, but secondary osteoarthritis, osteonecrosis, and local recurrence (12) are the main downsides. Although the latter shows low local recurrence rates, it comes with limitations such as limited prosthesis life, revision, infection, and poor joint function (13), especially for young patients.

Here in, we retrospectively analyzed cases of proximal femoral GCTB with complete clinical data through a single center. This study aims to study the clinical efficacy of EC and SR on proximal femoral GCTB and analyze the differences between the two surgical methods in terms of recurrence rate, functional reconstruction, postoperative complications, etc. The aim is to provide a theoretical basis for standardizing the treatment scheme and prospective research.

## Patients and Methods

### Patients

**Figure 1:**
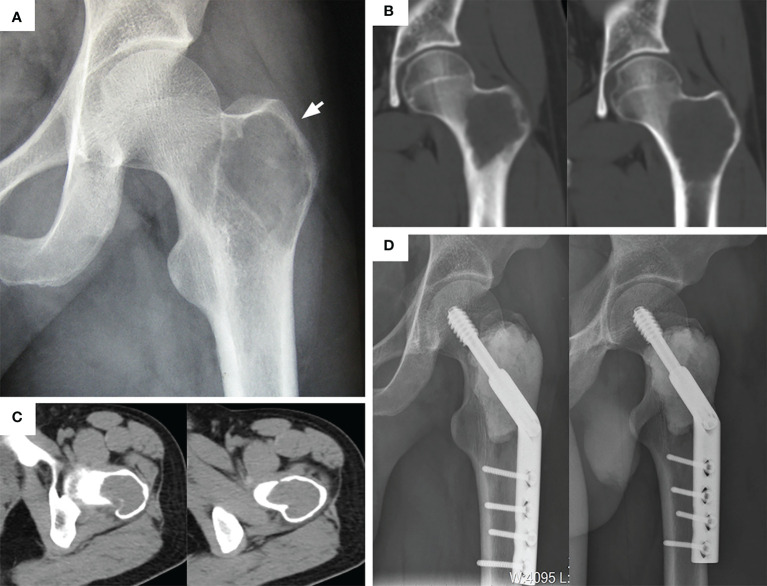
Typical preoperative and postoperative manifestations of EC for H2 type GCTB of proximal femur. **(A)** Apparent osteolytic lesions can be seen in the greater trochanter (arrow). **(B)** A coronal plane CT scan showed that the tumor invaded the femoral neck and intertrochanteric space. **(C)** CT transverse section showed that the bone cortex around the lesion was thin but not completely penetrated. **(D)** After extended curettage, allogeneic bone and bone cement filling, and DHS plate fixation. The anteroposterior and lateral radiographs was rechecked at 29 months.

From February 2010 to June 2018, 37 consecutive patients with a diagnosis of GCTB of the proximal femoral were treated at the Xiangya Hospital Bone Tumor Center. In this retrospective study, the inclusion criteria were: (1) the lesion was located in the proximal femur and confirmed as GCTB by histopathological diagnosis; (2) GCTB patients who were initially treated in the bone tumor treatment center of our hospital and undergone a primary operation; (3) and postoperative follow-up of more than 24 months with integrated data. The exclusion criteria were: (1) Presence of primary or secondary malignant giant cell tumor of bone (once the preoperative imaging data show that the tumor may deteriorate, we would take preoperative puncture biopsy to determine the diagnosis); (2) patients hospitalized for local recurrence or complications after treatment in other hospitals. According to the above criteria, among the 37 patients, 2 patients developed malignant changes, 4 were lost to follow-up, and 2 were admitted to our department due to postoperative complications after treatment in other hospitals. Finally, a total of 29 patients were included in this study. The localization of the lesion was performed using the International Society of Limb Salvage (ISOLS) zoning method: the tumor located in the femoral head was identified as the H1 zone, those between the femoral head and neck junction and the distal plane of the lesser trochanter as the H2 zone (**Figure 1**), and those in the distal plane of the lesser trochanter as the H3 zone (14). In addition, preoperative X-ray, computed tomography(CT), and magnetic resonance imaging(MRI) were used to evaluate the scope of tumor invasion, record whether pathological fracture and displacement were present, and Campanacci imaging grade was used to evaluate its performance (15). All the above patients and their guardians have signed informed consent. This study has been approved by the Ethics Committee of Xiangya Hospital of Central South University.

### Surgical Technique

**Figure 2:**
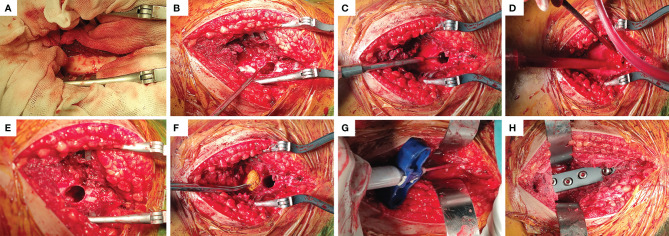
Main steps of EC surgery. **(A)** Fenestration was performed in the lateral position near the lesion. **(B)** Complete removal of the tumor tissue visible with curettes of different sizes. **(C)** The bone ridge in the tumor cavity was removed with a high-speed grinding drill. **(D)** the tumor cavity was flushed with a high-pressure sterilization water gun. **(E, F)** The cavity wall was wiped with a cotton ball soaked with 10% Iodine tincture. **(G)** Allogeneic bone was implanted into the subchondral bone with a thickness of at least 1cm, and the rest was filled with bone cement. **(H)** Driving DHS steel plate to stabilize mechanical stress.

The following procedure was used: for EC operation, a longitudinal incision was taken at the lateral side of the proximal thigh, with the tensor fascia lata and lateral femoral muscle membrane cut. This fully exposes the lesion (**Figure 2A**), and a bone drill was used to drill holes along the periphery of the fenestration at the proximal femur. To prevent splitting fractures, the fenestration should be sufficiently large to remove the tumor tissue completely. Curettes of different sizes (**Figure 2B**) were used, and the surrounding bone ridge was cleaned with a high-speed grinding drill (**Figure 2C**). The cavity wall was cauterized with a high-frequency electric knife. The tumor cavity was flushed with a high-pressure sterilization water gun (**Figure 2D**). A 10% Iodine tincture was applied meticulously using a surgical cotton ball and left for at least 1 minute (**Figures 2E, F**) to eliminate residual tumor cells. The surface of subchondral bone was filled with allogeneic bone (**Figure 2G**), then the main nail was implanted, and the remaining bone defect was filled with bone cement. It is worth noting that the allogeneic bone was filled below the subchondral bone with a thickness of at least 1 cm. Finally, with the assistance of a C-arm machine, the dynamic hip screw (DHS) steel plate was accurately inserted (**Figure 2H**), washed with normal saline, and the wound was closed. The procedure of SR operation was as follows: The posterolateral approach of the hip was used, the tumor boundary was fully exposed, and the soft tissue within 1 cm outside the tumor capsule and bony tissue within 2~3cm were removed entirely to achieve marginal resection. Healthy soft tissues were retained during the resection process, especially the lateral femoral muscle, to cover the prosthesis. A distal osteotomy was performed 2~3 cm away from the tumor. The anterior soft tissue was separated with dislocation of the femoral head while protecting the sciatic nerve, and the tumor segment was wholly removed. Measure the bone length of the excised segment, reconcile the bone cement, and a customized femoral prosthesis was inserted. The intercondylar connecting plane and the thick line of the femur were used as a reference to control the rotation of the prosthesis so that the femoral neck was tilted forward by 15°.

Patients in the EC group avoided weight-bearing for 2 weeks after the surgery and gradually transitioned from non-weight, semi-weight bearing to full-weight bearing with the support of crutches. Patients in the SR group began non-weight-bearing hip flexion and extension in bed 3 days after the surgery, semi-weight bearing and hip function exercises began 1-week post-surgery, semi squatting was practiced with the aid of crutches 3 weeks after surgery, and achieved normal life function score within 3 months.

### Follow-Up and Evaluation

The first reexamination was started in the first month after surgery, and follow-ups were conducted every 3 months in the first year after surgery, every 6 months in the second year, and then yearly, largely as outpatient follow-ups. The follow-up examinations included local X-rays, CT or MRI, and other routine auxiliary examinations. For those with lung metastasis before operation, we usually recheck chest CT every three months after operation. For those without lung metastasis before operation, we usually recheck every six months after operation. In addition, the functional status of the hip joint on the affected side was thoroughly checked with the aim to assess the postoperative tumor prognosis, functional prognosis, and complications. The evaluation methods were as follows: Kellgren-Lawrence (K-L) grade (16) was used to evaluate the severity of osteoarthritis, Musculoskeletal Tumor Society (MSTS) score (17) was used to evaluate the functional changes, Ficat classification (18) was used to monitor the status of femoral head necrosis, and Mankin evaluation standard (19) was used to evaluate the surgical efficacy. In addition, infection, prosthesis loosening, immune rejection, fracture, and recurrence were recorded, and the latest follow-up was to be taken as the final recorded.

### Statistical Analysis

SPSS version 20 (SPSS Inc., Chicago, IL, USA) was used to analyze the collected data, determine the relationship between different variables, and compare the therapeutic effects and prognostic outcomes of the two operations. The quantitative data were expressed as mean ± standard deviation, and conform to normally distributed. The difference of mean between the two groups was analyzed by independent sample t-test. Chi-square test or Fisher’s exact test were used to analyze the qualitative data expressed in frequency. P < 0.05 was deemed as statistically significant.

## Results

**Figure 3:**
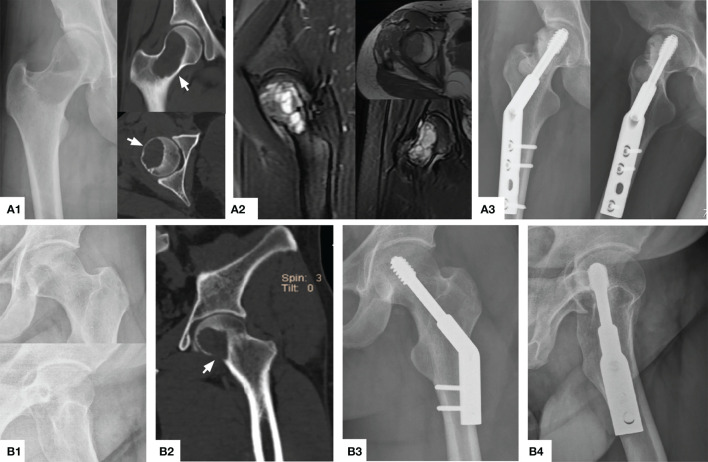
Typical preoperative and postoperative manifestations of EC for H1+H2 type GCTB of proximal femur. **(A1)** A 40 year female patient with osteolytic changes occurred in the whole femoral neck extending upwards to the femoral head (arrow). **(A2)** MRI showed that most of the lesions were medium to high-intensity signals without the involvement of the surrounding soft tissue. **(A3)** The X-ray showed that the bone graft was satisfactory and the internal fixation was firm 26 months after operation. **(B1, B2)** A 31 year male patient with obvious quasi-circular transparent area can be seen under the femoral head, accumulating down the femoral neck, and partial perforation of the bone cortex can be seen (arrow). **(B3, B4)** The bone healed satisfactorily and effective internal fixation 32 months after operation.

**Figure 4:**
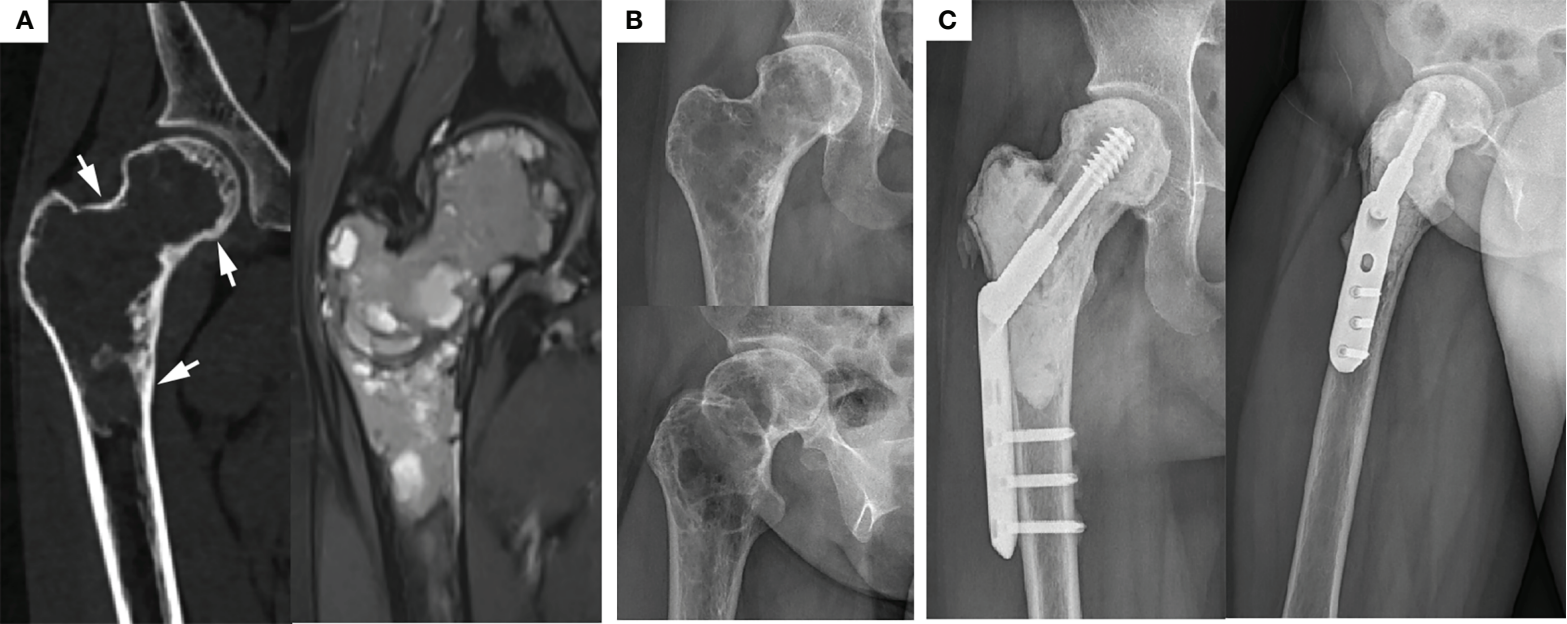
Typical preoperative and postoperative manifestations of EC for H1+H2+H3 type GCTB of proximal femur. **(A)** Preoperative CT and MRI showed that the femoral head, femoral neck, and subtrochanteric were invaded, but the lesions were still wrapped in the bone cortex (arrow). **(B)** Preoperative X-ray showed typical “soap bubble-like” changes. **(C)** Due to the extensive involvement of the lesion and the significant reduction of bone strength, the allogeneic fibula was placed in parallel above DHS and achieved a desirable prognosis 3 years after operation.

According to the original data of patients (**Table 1**), there were 19 patients (11 men and 8 women) in the EC group, with an average age of 32.3 years (range, 19-52). This group included 10 cases of Campanacci grade II and 9 cases of Campanacci grade III. 12 cases were located in the H2 area (**Figure 1**), 6 cases in the H1 + H2 area (**Figure 3**), 1 case in H1 + H2 + H3 area (**Figure 4**), and 2 cases had pathological fractures before the procedure. On the other hand, there were 10 patients (4 men and 6 women) in the SR group, with an average age of 31.8 years (range, 22-49). This group included 1 case of Campanacci grade II, 9 cases of Campanacci grade III. 1 case was located in the H1 area, 5 cases in the H2 area, 4 cases in the H1 + H2 area, and 8 cases had pathological fractures before the procedure. Besides, in all patients, no tumor cells were found in the adjacent tissues selected during the operation.

**Table 1:** Demographic and clinical follow-up data of patientBC, bone cement; AB, allogeneic bone; EC, extended curettage; SR, segmental resection; MSTS, musculoskeletal tumor society.

Patients Number/Gender	Age/Location	FillerMaterials	Disease Course (Month)	Therapeutic Modalities	Follow-up(Month)	Campanacci Grade	Pathological Fracture	Post-op MSTS Score	Post-Op recurrence	Complications
1/F	40/H1+2	BC +AB	9	EC	119	II	N	25	N	osteoarthritis
2/M	24/H2	BC	6	SR	86	II	Y	28	N	N
3/M	31/H1+2+3	BC+AB	12	EC	74	II	N	30	N	N
4/M	49/H1+2	BC	8	SR	52	III	Y	22	N	joint stiffness
5/F	29/H2	BC+AB	15	EC	91	III	Y	27	Y	N
6/M	25/H1+2	BC	18	SR	46	III	N	28	N	N
7/F	48/H2	BC	11	SR	61	III	Y	23	N	infection
8/F	41/H2	AB	17	EC	37	II	N	29	N	N
9/M	39/H1+2	BC+AB	18	EC	137	III	N	27	N	N
10/M	52/H2	BC+AB	5	EC	44	III	N	28	N	N
11/F	22/H1	BC	10	SR	121	III	N	21	N	Prosthesis loosening
12/M	27/H2	AB	6	EC	26	II	N	29	N	N
13/M	19/H2	AB	15	EC	57	II	Y	27	N	N
14/F	30/H1+2	BC	21	SR	65	III	Y	26	N	N
15/F	34/H2	BC	11	SR	40	III	Y	25	N	N
16/F	36/H2	AB	14	EC	41	II	N	27	N	N
17/F	24/H2	BC	17	SR	125	III	Y	21	Y	joint stiffness
18/M	33/H2	BC+AB	20	EC	28	III	N	30	N	N
19/F	21/H2	AB	7	EC	36	II	N	29	N	N
20/M	32/H1+2	BC	13	SR	27	III	Y	27	N	N
21/M	27/H2	BC+AB	16	EC	44	III	N	28	N	N
22/F	30/H2	BC	9	SR	36	III	Y	26	N	joint stiffness
23/M	38/H1+2	BC+AB	7	EC	50	III	N	28	N	N
24/M	31/H1+2	BC+AB	10	EC	31	III	N	29	N	N
25/F	28/H2	AB	19	EC	38	II	N	28	N	N
26/F	40/H2	AB	18	EC	47	II	N	26	N	N
27/M	37/H1+2	BC+AB	6	EC	79	III	N	24	N	osteonecrosis
28/F	24/H1+2	BC+AB	15	EC	50	III	N	29	N	N
29/M	20/H2	AB	17	EC	63	II	N	27	N	N

BC, bone cement; AB, allogeneic bone; EC, extended curettage; SR, segmental resection; MSTS, musculoskeletal tumor society.

### Oncology Prognosis

In this study, the average follow-up time of the EC group was 57.5 months (range, 26-137). Among the 19 patients, 1 patient (5.3%) developed local soft-tissue recurrence 17 months after surgery: this patient had a pathological fracture without apparent displacement before surgery and was treated with expanded curettage in consideration of the patient’s young age. In the second surgery, local resection was carried out, and the healing and recovery were fair. The follow-up results were satisfactory after 74 months. Another case had lung metastasis before surgery, but there were no secondaries after resection of pulmonary nodules under endoscopy, and tumor-free survival was achieved.

The average follow-up time of the SR group was 65.9 months (range, 27-125). Of the 10 patients, 1 patient (10%) developed local recurrence of the distal part of the prosthesis and was located in the proximal femur, 2 years after surgery. This was confirmed by pathological biopsy as GCTB. Therefore, tumor segment resection and artificial prosthesis construction were performed again. There was no recurrence and metastasis after 8 years of follow-up. Unfortunately, this patient suffered from local hip joint functional impairment due to two major invasive operations. In addition, there were no lung metastases in this group of patients before or after surgery.

In general, only 1 patient (5.3%, 10%) in both groups had a relapse, and the recurrence rate was not statistically different (P=1.000). Besides, results of the univariate analysis showed no significant correlation between gender, pathological fractures, surgical methods, lesion locations, Campanacci grades, and the recurrence of proximal femur GCTB (**Table 2**).

**Table 2:** Data statistics and analysis of patients.

Variable	EC group (n = 19)	SR group (n = 10)	P-value
Mean age, (sd)	32.3 ± 8.5	31.8 ± 9.6	
Gender, n (%)			
M	11	4	
F	8	6	
Campanacci Grade, n (%)			
II	10	1	
III	9	9	
location			
H1	0	1	
H2	12	5	
H1+2	6	4	
H1+2+3	1	0	
Pathological fracture, n (%)	2	8	<0.0001
Disease course(month)	12.9 ± 5.0	12.4 ± 4.8	
Duration of follow-up (month)	57.5 ± 30.4	65.9 ± 34.4	0.779
Local recurrence, n (%)	1 (5.3%)	`1 (10%)	1.000
Post-op MSTS score	27.6± 1.6	24.7± 2.8	0.002
Complication, n (%)			
osteoarthritis	1	0	
joint stiffness	0	3	
infection	0	1	
osteonecrosis	1	0	
Prosthesis loosening	0	1	
total	2 (10.5%)	5 (50%)	0.03
Reoperation, n (%)	1 (5.3%)	2 (20%)	
Excellent and good rate	18 (94.7%)	8 (80%)	0.560

EC, extended curettage; SR, segmental resection; MSTS, musculoskeletal tumor society.

### Function and Treatment Evaluation

The MSTS scoring system of bone and soft tissue tumors was used as a reference for postoperative functional evaluation. The EC group had an average score of 27.6 (range, 24-30), while the SR group had an average score of 24.7 (range, 21-28). Results of the statistical analysis identified that the EC group obtained better postoperative functional recovery than the SR group (P = 0.002). In addition, according to the Mankin evaluation standard, the surgical effect was comprehensively evaluated, and the excellent and good rates were calculated. In the EC group, 17 cases were rated as excellent, 1 was rated as good, and 1 was poor. In contrast, in the SR group, 8 cases were rated as excellent, and 2 were rated as poor; The overall excellent and good rates of the two groups were compared (EC group, 94.7%; SR group, 80%), EC group was slightly higher (P = 0.560) (**Table 2**).

### Complications

Among the 19 patients in the EC group, 1 patient developed hip arthritis (K-L grade 2) at the 102 month of postoperative follow-up but with no apparent pain and joint deformities, currently under conservative treatment. Another patient developed necrosis of the femoral head (Ficat stage I). Although the articular surface was not involved before the operation, the tumor invaded the subchondral bone of the femoral head in a wide range. Therefore, the local blood supply under the femoral head might have been affected during the extended curettage. Non-steroidal anti-inflammatory drugs and drugs to improve local microcirculation (prostaglandin E1) were temporarily given, the course of prostaglandin E1 was 3 months, 5ug/day, 14 days/month. In contrast, 3 patients developed varying degrees of joint stiffness in the SR group, and satisfactory results were obtained after standardized functional rehabilitation training. In addition, 1 patient suffered from a peri-prosthetic delayed infection at 5 months postoperatively, which was well-controlled after debridement, lavage, and drainage. Another patient suffered from a slight loosening of the prosthesis, but this did not affect the routine work and life of the patient. Due to financial reasons, the patient refused active treatment and continued with regular follow-up. Overall, the incidence of complications in the SR group (50%) was higher than that in the EC group (10.5%), the comparison being statistically significant (P = 0.03) (**Table 2**).

## Discussion

Proximal femoral GCTB, as an intermediate tumor with low incidence, local invasiveness, and strong bone destructiveness, can easily cause puncture of the cortical bone and pathological fractures (20). With the development of surgical technology and the improvement of adjuvant therapy, open surgery is the most effective treatment for most patients with GCTB. Extended curettage and segmental tumor resection are often used in clinical practice (10, 11). Still, even in the most commonly seen cases of GCTB of the knee joint, when combined with pathological fractures or Campanacci grade III, the choice of the two surgical methods remains controversial (20, 21). There are even fewer systematic studies for GCTB of the proximal femur to clarify the reference criteria for surgical selection.

In the past, due to the insufficient resection edge of the tumor, the recurrence rate of curettage and bone grafting was as high as 40%-60% (2, 22). Now, with the continuous improvement of the understanding of the invasiveness of GCTB, some scholars put forward the concept of extended curettage, using high-speed grinding and drilling to remove the invaded bone in the lesions. Pulse washing and application of chemical agents (phenol, alcohol, Iodine tincture, or zinc chloride) were used to further treat the tumor cavity to reduce postoperative recurrence rate (23, 24). Iodine tincture with a concentration of 10%, which can denature the cell membrane of tumor cells and induce coagulative necrosis. It has slight irritation to the solid substance of bone, so it plays an ideal role as a local tumor killer. In this study, after high-speed drilling, electric knife cauterization, pulse sterilized water, and iodophor smearing, the recurrence rate (5.3%) was effectively controlled. It was slightly lower than the extensive data research of our department (7.2%) (3) and significantly better than other single-center retrospective studies (25, 26). Moreover, the commonly used reconstruction materials after GCTB extended curettage include autologous bone, allogeneic bone, or bone cement (27, 28). Bone cement with good mechanical stress was used to fill the tumor cavity’s primary body reconstruction. Furthermore, bone cement can dissipate a lot of heat during solidification and physically inactivate the residual tumor cells around the tumor cavity. However, some studies have reported that using bone cement only to fill the expanded bone defect after the scraping promotes thermal injury of articular cartilage and non-fusion of the cement-subchondral bone interface (29). Radev BR et al. (30) recommend allogeneic bone transplantation (at least 3mm thick) at the subchondral bone to avoid this complication. This surgical technique also coincides with our study: when reconstructing bone defects, the subchondral bone was first filled with allogeneic bone, usually 10mm in thickness, and soaked with hydrogen peroxide before use, to remove its immunogenicity. Finally, bone cement is supplemented. Only 1 case in the EC group developed hip arthritis without surgery and obtained a satisfactory prognosis through the above multi-dimensional treatment methods.

Enlarged curettage of proximal femoral GCTB increases the risk of pathological fracture of the femoral neck. Errani C (31) and Lun D et al. (32) believe that the maximum diameter of the lesion shown by imaging exceeds 50% of the femoral neck, and the mechanical strength may be damaged, the bone cortex is involved in an extensive range, and may be further damaged during tumor curettage, resulting in pathological fractures. In the above cases, preventive internal fixation is required. In this group, 17 patients were treated with prophylactic DHS internal fixation, 2 patients with pathological fractures were reconstructed directly with DHS, and postoperative fractures were not observed. The trabecular bone pores at the proximal tuberosity of the femur are larger, and the tumor invasion may be more extensive, which indicates a more thorough removal. However, the bone of the femoral calcar is dense, which significantly impacts the mechanical strength of the proximal femur after destruction, this requires more attention during reconstruction. For proximal femoral GCTB, the tumor is first removed while preserving the joint, mechanical strength is then restored. If these conditions can be met at the same time, EC surgery should be chosen.

**Figure 5:**
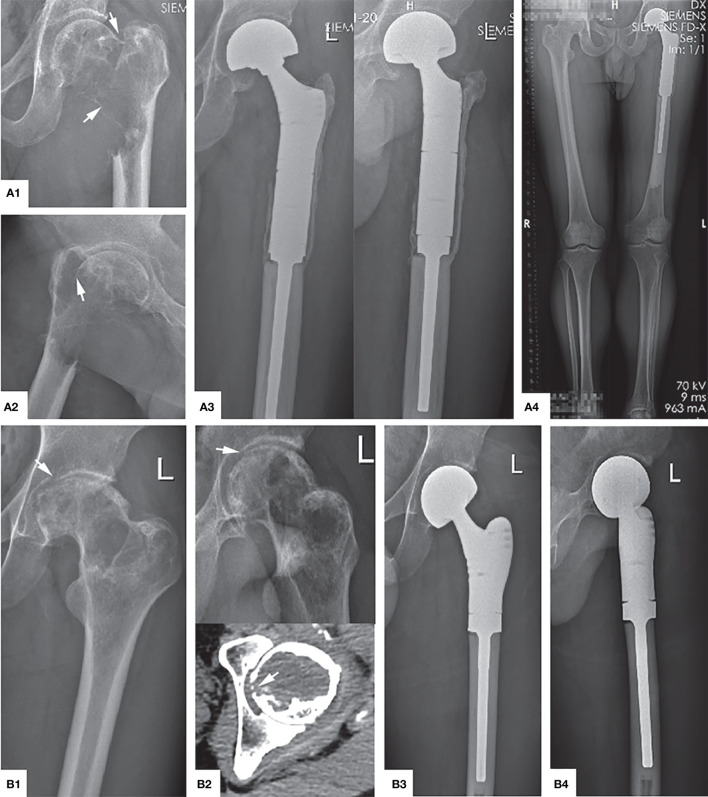
Main indications and typical preoperative and postoperative manifestations of SR. **(A1, A2)** The tumor has completely eroded the proximal femur, and a significantly displaced pathological fracture has occurred (arrow), which is Campanacci grade III. **(A3, A4)** Anteroposterior, lateral, and full-length of both lower limbs radiographs showed that the position of the prosthesis was adequate, there was no transparent band around, and the force line was normal 4 years after operation. **(B1)** The femoral head is compressed to flat due to osteolysis destruction (arrow). **(B2)** The articular surface was also damaged and ruptured (arrow). **(B3, B4)** The X-ray shows the contraposition and alignment of the artificial joint prosthesis were satisfactory 41 months after operation.

SR is a surgical method of segmental resection of the tumor, mega prosthesis implantation, and reconstruction. The indications for SR in this study include pathological fractures with evident displacement or Campanacci grade III proximal femoral GCTB while disrupting the integrity of the articular surface of the femoral head (**Figure 5**). SR is recommended for its excellent tumor prognosis. Van der Heijden L et al. (33) reported that for GCTB with pathological fractures, the local recurrence rate in SR was significantly lower than that of EC. Hindiskere S. et al. (34) and Klenke FM et al. (35) summarized multi-institutional retrospective studies, according to their experience, consider that SR has unique advantages in controlling the local recurrence rate of GCTB, SR is suitable for cases with a massive invasion of surrounding soft tissue. Interestingly, Balke M et al. (36) found that SR remains a wise choice for recurrent or worsening GCTB since it can effectively control the local recurrence rate. Therefore, SR is still a valuable treatment for high-grade and highly aggressive GCTB of the proximal femur. However, with advanced surgical techniques and treatment possibilities, preservation of joint function is preferred. The complications of SR, such as decreased limb functions, postoperative infection, long-term prosthesis loosening, and sinking, cannot be overlooked. Besides, it destroys the original joint structure while creating a large surgical wound and is associated with more intraoperative and postoperative bleeding, which hamper the target of optimal functional prognosis.

It was reported that aseptic loosening and prosthesis infection are the main reasons for the failure of prosthesis reconstruction after proximal femoral tumor (37). Xu G et al. (38) performed SR plus custom-made prosthesis reconstruction on 19 patients with proximal femoral tumors. 26% of the patients suffered from complications: 2 cases needed prosthesis removal, 2 cases developed deep infections around the acetabulum, and 2 cases developed acetabular wear. Abou Senna WG et al. (39) reported the complication rate after prosthesis replacement for proximal femoral tumors as 45%. The most common complication was periprosthetic infections in 10 cases (16.7%), followed by aseptic loosening in 7 cases (11.7%). These studies suggest that with the extension of survival and follow-up time, patients with SR plus prosthesis replacement will likely face many complications, leading to secondary revision surgery. In addition, the joint functions will be gradually lost, increasing the economic burden of patients, and it remains tough to obtain a satisfactory functional prognosis at the same time. These factors must be considered during the initial SR operation.

The indications of SR and EC are different, but their long-term outcome can be compared in order to find the balance point in the treatment of proximal femoral GCTB. This study’s analysis and comparison established that the recurrence rate between the EC group (5.3%) and SR group (10%) was similar. Interestingly, there was no statistical difference, suggesting that EC can also obtain a satisfactory local control rate. However, in terms of functional recovery, the MSTS score of the EC group (27.6 ± 1.6) was significantly higher than that of the SR group (24.7 ± 2.8). Meanwhile, the incidence of complications in the EC group (10.5%) was significantly lower than that in the SR group (50%), with both groups achieving satisfactory excellent and good rates (EC versus SR, 94.7% versus 80%). Summing up the above results, there are substantial differences in the long-term functional prognosis and complication rates between EC and SR, which must be regarded as an essential factor in surgical decision-making. In addition, with the emergence of microwave ablation and denosumab adjuvant therapy, both may downgrade the surgery so that more patients can receive EC surgery (6, 24). In short, the author believes that EC can effectively control the local recurrence rate and obtain ideal postoperative function for patients with Campanacci grades II and III without extensive soft tissue invasion or pathological fracture without evident displacement. On the other hand, SR is more suitable for patients with GCTB of the proximal femur with damaged articular surfaces that cannot be preserved or from pathological fractures with obvious displacement.

This study is a single-center retrospective analysis but contains some shortcomings: (1) due to the low incidence rate of GCTB in the proximal femur, the total number of cases in this study is relatively small, therefore, larger samples and more extensive data analysis are required in the future; (2) Although all follow-up time were > 2 years, the duration needs to be extended for the analysis of the long-term survival rate of artificial joint prostheses.

## Conclusion

In conclusion, functional reconstruction and recurrence control play a vital role for GCTB in the proximal femur. When the tumor does not extensively involves the surrounding soft tissues, the articular surface is not damaged, and there is no pathological fracture with apparent displacement, EC should be fully considered to achieve optimal joint function and survival prognosis, In other cases, SR surgery is also a wise choice.

## Data Availability Statement

The original contributions presented in the study are included in the article/supplementary material. Further inquiries can be directed to the corresponding author.

## Ethics Statement

All patients and their families provided informed consent, and the study design was approved by the Research Ethics Committee of Xiangya Hospital.

## Author Contributions

YY and WL were in charge of the study design. HH, WL, QL, YL, WZ, and YY screened patients and collected relevant follow-up data. YY, QL, ZW, and YL analyzed the data. The manuscript was written by YY and WL. WL checked the manuscript. All authors contributed to the article and approved the submitted version.

## Funding

This work was supported by the Hunan Youth Science and Technology Innovation Talent Project (2020RC3058), and the Research project of Hunan health and Family Planning Commission (C20180785).

## Conflict of Interest

The authors declare that the research was conducted in the absence of any commercial or financial relationships that could be construed as a potential conflict of interest.

## Publisher’s Note

All claims expressed in this article are solely those of the authors and do not necessarily represent those of their affiliated organizations, or those of the publisher, the editors and the reviewers. Any product that may be evaluated in this article, or claim that may be made by its manufacturer, is not guaranteed or endorsed by the publisher.

## Abbreviations

EC, extended curettage; SR, segmental resection; GCTB, giant cell tumor of the bone; ISOLS, International Society Of Limb Salvage; MSTS, musculoskeletal tumor society; CT, computed tomography; MRI, magnetic resonance imaging; DHS, dynamic hip screw.
